# Dissociation Between *APOC3* Variants, Hepatic Triglyceride Content and Insulin Resistance

**DOI:** 10.1002/hep.24072

**Published:** 2011-02

**Authors:** Julia Kozlitina, Eric Boerwinkle, Jonathan C Cohen, Helen H Hobbs

**Affiliations:** 1 McDermott Center for Human Growth and Development, University of Texas Southwestern Medical CenterDallas, TX; 2 Howard Hughes Medical Institute, University of Texas Southwestern Medical CenterDallas, TX; 3 Human Genetics Center and Institute for Molecular Medicine, University of Texas Health Science CenterHouston, TX

## Abstract

Nonalcoholic fatty liver disease (NAFLD) is an escalating health problem that is frequently associated with obesity and insulin resistance. The mechanistic relationship between NAFLD, obesity, and insulin resistance is not well understood. A nonsynonymous variant in patatin-like phospholipase domain containing 3 (rs738409, I148M) has been reproducibly associated with increased hepatic triglyceride content (HTGC) but has not been associated with either the body mass index (BMI) or indices of insulin resistance. Conversely, two sequence variants in apolipoprotein C3 (*APOC3*) that have been linked to hypertriglyceridemia (rs2854117 C > T and rs2854116 T > C) have recently been reported to be associated with both hepatic fat content and insulin resistance. Here we genotyped two *APOC3* variants in 1228 African Americans, 843 European Americans and 426 Hispanics from a multiethnic population based study, the Dallas Heart Study and test for association with HTGC and homeostatic model of insulin resistance (HOMA-IR). We also examined the relationship between these two variants and HOMA-IR in the Atherosclerosis Risk in Communities (ARIC) study. No significant difference in hepatic fat content was found between carriers and noncarriers in the Dallas Heart Study. Neither *APOC3* variant was associated with HOMA-IR in the Dallas Heart Study; this lack of association was confirmed in the ARIC study, even after the analysis was restricted to lean (BMI < 25 kg/m^2^) individuals (n = 4399). *Conclusion:* Our data do not support a causal relationship between these two variants in *APOC3* and either HTGC or insulin resistance in middle-aged men and women. (Hepatology 2011;53:467-474)

Nonalcoholic fatty liver disease (NAFLD) is a highly prevalent condition estimated to afflict approximately one-third of the adult population in Western countries.^1^ NAFLD comprises a spectrum of hepatic disorders extending from hepatic steatosis to cirrhosis.^2^, ^3^ The hallmark of hepatic steatosis is the presence of triglycerides (TGs) stored as lipid droplets in the cytoplasm of hepatocytes. It has been estimated that 10% to 20% of those with steatosis develop inflammation (steatohepatitis), and the disease can progress to cirrhosis and perhaps to hepatocellular carcinoma.^3^ It is anticipated that NAFLD will soon overtake hepatitis C as the most common indication for liver transplantation.

A major factor contributing to the increase in hepatic triglyceride content (HTGC) in the general population is the high prevalence of obesity and insulin resistance. Hepatic TG levels typically are low in lean, nondiabetic individuals, whereas approximately 50% of those with a body mass index (BMI) > 30 kg/m^2^^1^ and approximately 75% of those with adult-onset type 2 diabetes have steatosis.^3^ Thus, although obesity and insulin resistance are important susceptibility factors for NAFLD, not all obese or insulin-resistant individuals develop hepatic steatosis. The mechanisms underlying the variation in susceptibility to NAFLD and the causal nature of the relationships between obesity, hepatic TG, and insulin resistance are poorly understood.

Genetic association provides a powerful tool for dissecting the mechanistic relationships between variables that are correlated in complex diseases. In 2008, a single-nucleotide polymorphism (SNP) in the patatin-like phospholipase domain containing 3 protein 3 encoding gene (*PNPLA3*) was identified that was strongly associated with HTGC in Caucasians, Hispanics, and African Americans.^4^ The variant (rs738409) results in the substitution of methionine for isoleucine at residue 148 of PNPLA3. The PNPLA3-I148M variant is most common in Hispanics [minor allele frequency (MAF) = 0.48], the group with the highest prevalence of hepatic steatosis (45%), and is least common in African Americans (MAF = 0.14), who have the lowest incidence of steatosis (24%); the frequency in Caucasians is intermediate (MAF = 0.23). Hispanics who are homozygous for the variant have an approximately 2-fold increase in HTGC, whereas African American and European American homozygotes have 60% and 30% increases in median HTGC, respectively. According to the homeostatic model assessment of insulin resistance (HOMA-IR), despite the clear association between the variant and HTGC, PNPLA3-I148M is not associated with insulin resistance in either the Dallas Heart Study or the Atherosclerosis Risk in Communities (ARIC) study.^4^ The variant was independently identified in a genome-wide association study of serum liver enzyme levels.^5^ Subsequent studies in other populations have confirmed that PNPLA3-I148M is associated with increased liver fat content and elevated plasma levels of aminotransferases but not with BMI, insulin sensitivity, or plasma TG levels.^4^, ^6^-^12^

Recently, two SNPs in the promoter region of the gene encoding apolipoprotein C3 [*APOC3*; rs2854117 (−482 C > T) and rs2854116 (−455 T > C)] were reported to be associated with an approximately 3-fold increase in median HTGC values and with insulin resistance.^13^ The two variants, which had previously been found to be associated with plasma TG levels in some studies,^14^-^16^ are located in a putative insulin response element^17^ located 5′ to exon 1 of *APOC3*. *In* *vitro* promoter studies have suggested that insulin binding to this site inhibits *APOC3* transcription. The two variants prevent insulin binding and thus increase the levels of APOC3 messenger RNA and protein.^17^ APOC3 is transported on circulating lipoproteins and limits clearance of TG-rich particles.^18^ Petersen et al.^13^ proposed that the sequence variants lead to increased uptake of chylomicron remnants by the liver, and this results in NAFLD and hepatic insulin resistance.

Here we examined the relationship between *APOC3* genotypes, HTGC, insulin resistance, and fasting TG levels in the Dallas Heart Study, a probability sample of Dallas County^19^ in which HTGC was measured noninvasively with proton magnetic resonance spectroscopy (^1^H-MRS).^1^, ^4^, ^20^ We also tested for the combined effect of the *PNPLA3* and *APOC3* variants. To confirm our findings, we analyzed the relationship between the two *APOC3* SNPs, fasting TG levels, and insulin resistance in the ARIC study.^22^ No association was found between either of the SNPs and indices of insulin resistance in the Dallas Heart Study or in the ARIC study.

## Patients and Methods

### Human Subjects

The Dallas Heart Study is a multiethnic population-based probability sample of Dallas County residents (African Americans self-identified as black, 52%; individuals of mixed European descent self-identified as white, 29%; Hispanics self-identified as Hispanic, 17%; and other ethnicities, 2%). The sampling design and the study protocol have been described previously.^19^ The study was approved by the institutional review board of the University of Texas Southwestern Medical Center at Dallas, and all participants provided written, informed consent. Fasting venous blood samples were obtained from 3551 individuals, 3071 of whom completed a clinic visit. Alcohol consumption (g/day) was determined from responses to previously validated questions (Institute for Survey Research, Temple University, Philadelphia, PA, 1996). HOMA-IR was calculated from the fasting plasma glucose and insulin values, which were measured as described.^21^

HTGC was measured with ^1^H-MRS.^20^ Localized spectra of the liver were obtained with a 1.5T Gyroscan Intera MR system (Philips Medical Systems, the Netherlands), and HTGC was calculated as described.^1^ Of the individuals who completed a clinic visit, 2349 underwent ^1^H-MRS. Some study participants failed to obtain an MRS study because of claustrophobia (n = 191), a medical contraindication (n = 49), equipment failure (n = 19), refusal (n = 74), or scheduling conflicts (n = 289). In addition, 58 extremely obese individuals (>145 kg) were excluded because of the weight limitations of the table.^1^ From the 2349 ^1^H-MRS measurements, data of sufficient quality to determine HTGC were obtained for 2239 individuals, who included 1104 African Americans, 734 European Americans, and 401 Hispanics. Hepatic steatosis was operationally defined as a liver fat content of 5.5% or greater, which corresponds to the 95th percentile of the liver fat content in lean, healthy individuals from the Dallas Heart Study.^20^ The definition is based on population prevalence and is not coupled to a future risk of steatohepatitis and cirrhosis.

#### Genotyping

Genomic DNA was extracted from circulating leukocytes. Genotypes for the rs2854117 (−482 C > T) and rs2854116 (−455 T > C) polymorphisms were determined with the TaqMan AD assay (Applied Biosystems). Oligonucleotides used for genotyping are shown in Supporting Table 1. A total of 3477 individuals from the Dallas Heart Study were successfully genotyped for the *APOC3* rs2854117 variant; they included 2952 participants who completed a clinic visit and 2198 subjects with HTGC measurements (1081 African Americans, 726 European Americans, and 391 Hispanics). A total of 3399 subjects were successfully genotyped for *APOC3* rs2854116, and these included 2880 participants who completed a clinic visit and 2150 individuals with hepatic TG measurements (1096 African Americans, 733 European Americans, and 401 Hispanics). Both genotypes were determined for 3336 of these subjects, who included 2113 with HTGC measurements.

The two *APOC3* SNPs were also genotyped in the ARIC study with the TaqMan AD assay (Applied Biosystems).

#### Inferred Ancestry

To account for a possible substructure in the self-identified ethnic groups, we inferred ancestry in the Dallas Heart Study participants with STRUCTURE^23^ under a linkage model with 2270 ancestry-informative SNPs^24^ as described.^4^

#### Statistical Analysis

Associations between SNP genotypes and HTGC, HOMA-IR, fasting TG, alanine aminotransferase (ALT), and aspartate aminotransferase (AST) were tested with linear regression models with age, gender, ethnicity, and BMI as covariates. The relationships were also assessed in each ethnic group separately. To avoid confounding by population stratification, we repeated the analysis with the inferred ancestry score as a covariate instead of self-reported ethnicity. We applied a power transformation (λ = 1/4) to HTGC, a natural log transformation to plasma insulin levels, HOMA-IR, and BMI, and a log-log transformation to TGs, ALT, and AST before the analysis to render the error distributions approximately normal. Diabetic individuals were excluded from the analysis. Alcohol consumption was included as a covariate when we were testing for an association with HTGC. Genotypes were coded as 0, 1, or 2, and the association was tested under the assumption of an additive model. Deviations from Hardy-Weinberg proportions were tested with the exact test for Hardy-Weinberg equilibrium. All statistical analyses were performed with the R statistical language (R Development Core Team, 2008).

#### Web Resources

The free software R can be downloaded from the Comprehensive R Archive Network (http://www.cran.r-project.org).

## Results

### Insulin Resistance and HTGC

**Fig. 1:**
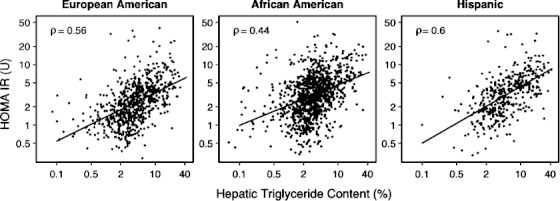
Relationship between HOMA-IR and HTGC with stratification by ethnicity in the Dallas Heart Study. The solid lines denote least squares regression lines.

The liver TG content was strongly correlated with HOMA-IR in the three major ethnic groups in the Dallas Heart Study (Fig. 1), as indicated by Spearman's rank correlation coefficient (ρ), a measure that is invariant to monotonic transformations of the data. Both HTGC and HOMA-IR were strongly associated with BMI in this sample, as previously reported.^1^ After adjustments for age, gender, ethnicity, and BMI, HTGC could account for 10% of the variation in HOMA-IR in the combined sample. BMI could account for approximately 15% of the variation in HOMA-IR after adjustments for age, gender, ethnicity, and HTGC (data not shown). Thus, neither HTGC nor BMI could explain the major fraction of variation in HOMA-IR.

#### Allele Frequencies

The frequencies of the *APOC3* polymorphisms differed markedly among ethnic groups. The variant alleles [rs2854117 (T) and rs2854116 (C)] were most common in African Americans (66.4% and 71.2%, respectively) and less common in Hispanics (32.7% and 38.9%, respectively) and Europeans (25.5% and 36.6%, respectively). These estimates are consistent with allele frequencies reported previously.^15^ Within each group, the genotype distributions were in Hardy-Weinberg equilibrium (Supporting Tables 2 and 3). The SNPs belonged to a larger linkage disequilibrium block spanning the *APOA5*/*APOA4*/*APOC3*/*APOA1* gene region (Supporting Fig. 1) and were in strong linkage disequilibrium (*D*′ = 0.98 and *R*^2^ = 0.75 in African Americans, *D*′ = 0.93 and *R*^2^ = 0.53 in European Americans, and *D*′ = 0.98 and *R*^2^ = 0.73 in Hispanics), as previously described.^14^

#### Genetic Association Between APOC3 and HTGC, HOMA-IR, and Plasma TG

**Fig. 2:**
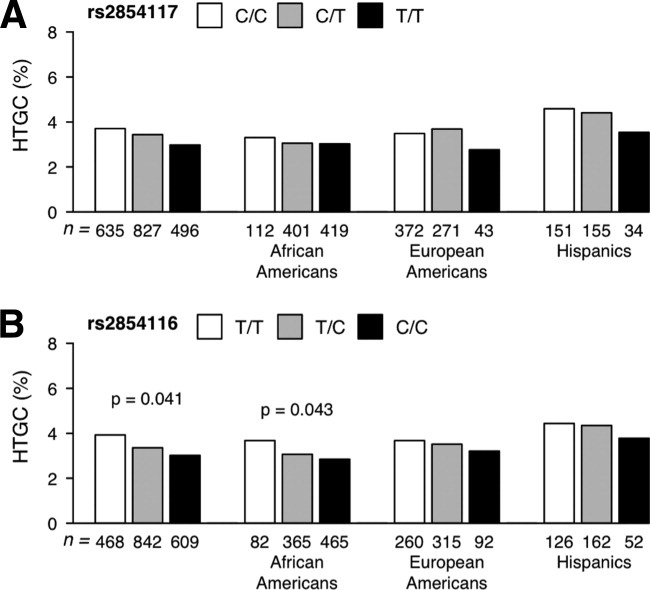
Median HTGC values in the Dallas Heart Study subjects stratified by ethnicity and genotype: (A) *APOC3* rs2854117 and (B) *APOC3* rs2854116. HTGC values were power-transformed (λ = 1/4) before the analysis, and *P* values were determined using a linear regression model with adjustment for age, gender, BMI, and alcohol consumption. Diabetics were excluded from the analysis.

**Fig. 3:**
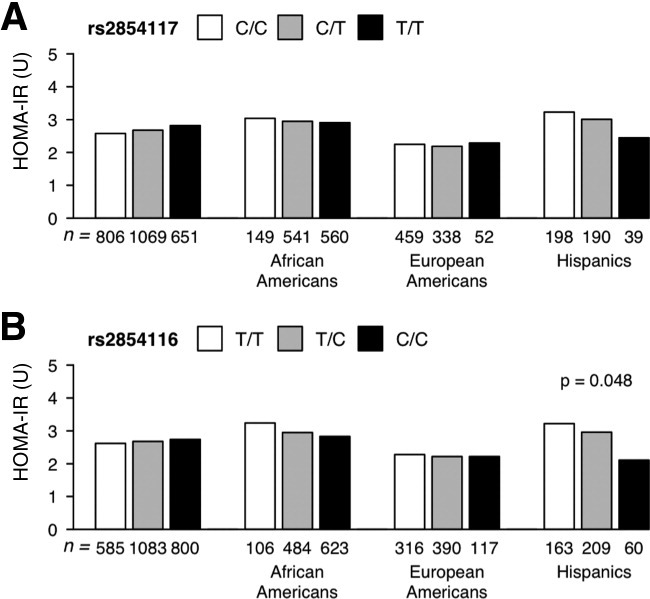
Median HOMA-IR values in the Dallas Heart Study participants stratified by ethnicity and genotype: (A) *APOC3* rs2854117 and (B) *APOC3* rs2854116. HOMA-IR values were log-transformed (natural logarithm), and *P* values were determined using a linear regression model with adjustment for age, gender, and BMI. Diabetics were excluded from the analysis.

First, we examined the relationship between each *APOC3* variant, HTGC, and HOMA-IR in the Dallas Heart Study. The *APOC3* rs2854117 (C > T) variant was not associated with HTGC in the overall sample or in any of the three ethnic groups (Fig. 2A). In contrast to the findings of Petersen et al.,^13^ the rs2854116 variant allele (C) was associated with a slight but significant reduction in HTGC (median HTGC: 4.1% in C/C genotype carriers versus 3.2% in T/T genotype carriers, *P* = 0.04; Fig. 2B). No association was found between either variant and the fasting levels of plasma glucose or insulin (Supporting Tables 2-5) or HOMA-IR except in Hispanics where the variant was marginally associated with a lower HOMA-IR (Fig. 3).

Neither *APOC3* sequence variant showed consistent relationships with plasma TG concentrations among the three ethnic groups. The rs2854117 (T) allele was associated with a modest increase in fasting TG concentrations in African Americans (median TG level: 78 mg/dL in C/C versus 84 mg/dL in T/T, *P* = 0.014; Supporting Table 2) but not in the other ethnic groups. rs2854116 was not associated with fasting plasma TG levels (Supporting Tables 3 and 5). No associations were found between either *APOC3* variant and the plasma levels of ALT or AST (Supporting Tables 2-5).

In some previous studies, the association between these two *APOC3* variants and plasma TG levels was apparent only in specific subgroups, such as healthy (nondiabetic) lean individuals^13^, ^16^ and nonsmokers.^15^ To investigate whether a relationship between *APOC3* variants and HTGC was apparent only in a subset of individuals or was obscured by smoking, we confined the analysis to the subset of Dallas Heart Study participants who were nondiabetic, had a BMI less than 25 kg/m^2^, and consumed less than 30 g of alcohol per day (n = 468), and we tested for an interaction with smoking status. No relationship between *APOC3* variants and HTGC or insulin resistance emerged in this subgroup (data not shown) in either men (n = 210) or women (n = 258). The results were not affected by smoking status.

**Fig. 4:**
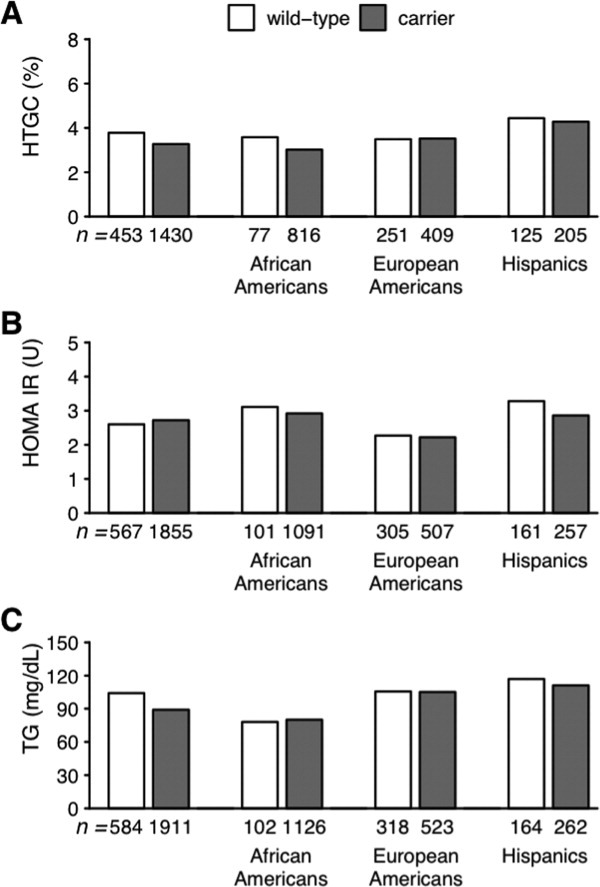
Median values of (A) HTGC, (B) HOMA-IR, and (C) plasma TG levels in noncarriers [individuals homozygous for the *APOC3* reference allele (rs2854117 C-482 and rs2854116 T-455)] and carriers (individuals with 482T, 455C, or both alleles) in the Dallas Heart Study.

We next investigated the possibility of a combined action of the *APOC3* polymorphisms through a comparison of the wild-type homozygotes (rs2854117 C/C and rs2854116 T/T) to carriers of one or more variant alleles [rs2854117 (T) and rs2854116 (C)], as was done previously.^13^ No significant relationships were observed between the two groups (carriers versus noncarriers for either variant) in HTGC, HOMA-IR, or fasting plasma TG levels (Fig. 4 and Tables 1 and 2).

**Table 1:** Clinical Characteristics of *APOC3* Wild-Type Homozygotes (rs2854117 C/C and rs2854116 T/T) and Carriers of One or More Variant Alleles (rs2854117 T and rs2854116 C) in the Dallas Heart Study

Characteristic	Wild-Type Homozygotes	Variant-Allele Carriers	*P* Value
n	586	1911	
Female/male	301/285	1071/840	
Age (years)	43.9 ± 9.9	43.8 ± 9.7	0.069
BMI (kg/m^2^)	29.3 ± 6.3	30.3 ± 7.4	0.986
Insulin (mIU/L)	11.7 (6.8-18.8)	11.8 (7.2-19.5)	0.3
Glucose (mg/dL)	92 (85-99)	91 (84-98)	0.522
HOMA-IR (U)	2.6 (1.5-4.3)	2.7 (1.6-4.5)	0.274
TGs (mg/dL)	104 (72-156.5)	89 (65-131)	0.398
HTGC (%)	3.8 (2.1-7.7)	3.3 (2.0-5.6)	0.175
AST (U/L)	21 (18-25)	21 (18-26)	0.376
ALT (U/L)	19 (15-28)	19 (14-27)	0.227

Means and standard deviations are presented for the age and BMI, and medians and interquartile ranges are presented for all other characteristics. *P* values were calculated with a linear regression model as described in the Patients and Methods section.

**Table 2:** Clinical Characteristics of Homozygotes for *APOC3* Wild-Type Alleles (rs2854117 C/C and rs2854116 T/T) and Carriers of Variant Alleles (rs2854117 T and rs2854116 C) in the Dallas Heart Study

	African Americans			European Americans			Hispanics		
			
Characteristic	Noncarriers	Carriers	*P* Value	Noncarriers	Carriers	*P* Value	Noncarriers	Carriers	*P* Value
n	102	1126		320	523		164	262	
Female/male	55/47	647/479		162/158	263/260		84/80	161/101	
Age (years)	45.5 ± 10.1	44.5 ± 9.7	0.332	45.1 ± 9.9	44.7 ± 9.7	0.575	40.7 ± 8.9	39 ± 8.3	0.039
BMI (kg/m^2^)	30.9 ± 7.3	31.2 ± 8	0.974	28.8 ± 6.3	28.6 ± 6.2	0.705	29.5 ± 5.2	30 ± 6.4	0.458
Insulin (mIU/L)	14.2 (8.7-22.0)	12.9 (7.7-20.7)	0.071	9.9 (6.0-16.4)	10.1 (6.0-16.6)	0.514	14.1 (8.5-20.5)	13.0 (7.6-19.8)	0.235
Glucose (mg/dL)	91 (84-99)	90 (83-97)	0.206	91 (84-97)	91 (83-97.5)	0.769	96 (87-101)	93 (87-100)	0.714
HOMA-IR (U)	3.1 (1.9-5.2)	2.9 (1.7-4.8)	0.059	2.3 (1.3-3.8)	2.2 (1.3-3.9)	0.534	3.3 (1.9-4.9)	2.9 (1.7-4.8)	0.244
TG (mg/dL)	78 (54.5-124)	80 (60-113)	0.312	105.5 (75-164)	105 (73-155.5)	0.975	117 (83-176)	111 (78-173)	0.446
HTGC (%)	3.6 (2.3-5.2)	3.0 (1.9-4.8)	0.106	3.5 (1.9-6.9)	3.5 (2.1-6.8)	0.771	4.4 (2.8-11.8)	4.3 (2.5-9.0)	0.34
AST (U/L)	20 (17-23)	21 (17-26)	0.108	21 (18-25)	21 (18-25)	0.827	21 (17-29)	21 (17-26)	0.927
ALT (U/L)	16 (12-23)	18 (13-26)	0.023	20 (15-27)	20 (16-27)	0.834	21 (15-33)	20 (15-32)	0.922

The subjects are stratified by ethnicity. Means and standard deviations are presented for the age and BMI, and medians and interquartile ranges are presented for all other traits. *P* values were calculated with a linear regression model as described in the Patients and Methods section.

Finally, to examine the combined effects of the *APOC3* SNPs and the *PNPLA3* rs738409 (C > G) polymorphism on HTGC, we fit a linear regression model including all three variants as predictors. The *PNPLA3* rs738409 risk allele (G) remained highly significant, even when the two *APOC3* variants were included in the model (*P* = 4.06 × 10^−13^). On the contrary, none of the *APOC3* SNPs were significantly related to HTGC with *PNPLA3* rs738409 in the model. In addition, we analyzed the relationship between *APOC3* variants and liver fat content in PNPLA3-148M carriers and in PNPLA3-148I homozygotes. No significant associations with HTGC were found in either group (data not shown). In the Dallas Heart Study, the *PNPLA3* rs738409 variant explained a variable proportion of HTGC in the three different ethnic groups, and this reflected the differences in allele frequencies between African Americans (MAF = 14%), European Americans (MAF = 23%), and Hispanics (MAF = 48%). The PNPLA3 polymorphism explains 12% of the variation in HTGC in Hispanics, 4% in African Americans, and only 2% in European Americans. Because African Americans comprised approximately 52% of the study participants, the PNPLA3 polymorphism explained 4% of the variation in HTGC in the entire Dallas Heart Study sample after adjustments for ethnicity, age, sex, BMI, and alcohol consumption. *APOC3* variants accounted for no meaningful variation in HTGC in any of the ethnic groups.

#### Confirmation Study

To confirm the prevalence of *APOC3* variants and the results of the association analysis, we examined the two SNPs in the ARIC study. HTGC was not measured in the ARIC study; therefore, we tested each variant for an association with fasting plasma TG levels and with HOMA-IR. Clinical characteristics of the ARIC population stratified by the *APOC3* genotype are shown in Supporting Tables 6 to 8. The frequencies of the *APOC3* rs2854117 and rs2854116 polymorphisms in the ARIC population were similar to those observed in the Dallas Heart Study in African Americans and Caucasians (Supporting Tables 6-8). The linkage disequilibrium block structure between the variants in the *APOA5*/*APOA4*/*APOC3*/*APOA1* gene cluster mirrored that calculated with the Dallas Heart Study data (Supporting Fig. 2). The variant allele at rs2854117 (T) was associated with higher plasma TG levels in ARIC whites (*P* = 0.001) but not in African Americans. Neither variant was significantly associated with HOMA-IR. No associations were observed when the analysis was confined to nondiabetic, lean (BMI < 25 kg/m^2^) individuals who consumed <30 g of ethanol per day (684 African Americans, including 329 men, and 3715 Caucasians, including 1167 men).

## Discussion

The goal of the present study was to elucidate the nature of the relationship between the liver TG content and insulin resistance through an examination of the metabolic sequelae of two sequence polymorphisms in *APOC3* that were associated with the liver TG content in a previous study.^13^ Here we found that one of the variants (rs2854116) was weakly associated with HTGC (*P* = 0.041) but in the direction opposite to that of the previous report.^13^ Carriers for this variant had lower HTGC values than noncarriers. The rs2854117 polymorphism had no detectable effect on levels of liver fat. Neither variant was associated with HOMA-IR in the Dallas Heart Study or in the ARIC study, even after the analysis was restricted to lean, nondiabetic individuals who consumed <30 g of ethanol per day. Thus, the minimal effect of the variants on the liver TG content and the lack of an effect on insulin resistance could not be explained by confounding due to ethnicity, obesity, or alcohol use. Taken together, our findings do not support a model in which a genetic polymorphism in the putative insulin response element of *APOC3* leads to increased accumulation of TG in the liver, which in turn results in insulin resistance.

Rather, our data are consistent with previous findings showing that genetic variation in *APOC3* expression has little effect on insulin sensitivity in humans^25^ or in mice.^26^

The reasons for the differences between our results and those of Petersen et al.^13^ are not clear. One possibility is that secondary factors such as obesity, exercise training, and alcohol use obscured the relationship between the *APOC3* polymorphism and insulin resistance in our population. However, the two polymorphisms were not associated with insulin resistance even when these factors were excluded. An alternative explanation is that the differences are due to ethnic differences in the two populations. The initial association between the sequence variations in *APOC3* and HTGC was identified in 95 Asian Americans, a group not represented in our study. It may be that the nucleotide substitutions on the variant allele of *APOC3* are not directly responsible for the associations with liver fat and insulin resistance observed in their Asian-Indian subjects but are in linkage disequilibrium with causal variants in this population that are not present in the ethnic groups (Caucasians, African Americans, and Hispanics) that we studied. However, Petersen et al. reported essentially identical findings in a second group composed mainly of Caucasian men. Therefore, it seems highly unlikely that differences in allele structure can account for the different effects of *APOC3* alleles observed in the two studies. Recently, Pollin et al.^25^ reported that 5% of the Lancaster Amish are heterozygotes for a null allele (R19X) of APOC3. Carriers of the mutation had a 50% reduction in circulating levels of APOC3, but their plasma glucose and insulin levels were indistinguishable from those of noncarriers. Thus, an approximately 50% reduction in circulating levels of APOC3 is not associated with changes in insulin sensitivity, at least in this population. Similarly, mice overexpressing APOC3 did not have insulin resistance despite markedly elevated plasma TG levels.^18^

The rs2854117 and rs2854116 variants were reported to be in linkage disequilibrium with a polymorphic *Sst*I site in the 3′ untranslated region of *APOC3* that was strongly associated with hypertriglyceridemia in several small studies.^14^, ^27^ In subsequent studies using larger samples, the association was inconsistent. Russo et al.^28^ reported that the *SstI* variant was not significantly associated with plasma TG levels in 1219 men and 1266 women in the Framingham Heart Study. Conversely, Waterworth et al.^29^ found a modest but statistically significant association between the *SSt*I polymorphism and plasma TG levels in 2745 Caucasian men from the Second Northwick Park Heart Study. In the present study, the rs2854117 polymorphism was significantly associated with plasma TG levels in 9799 Caucasian men and women from the ARIC study. The effect on plasma TG levels of the rs284117 variant and the *SstI* polymorphism was comparable in all three studies: homozygotes for the minor allele had plasma TG levels that were approximately 10% higher than those of homozygotes for the common allele. Thus, a single allele altered plasma TG levels by approximately 5%. In contrast, the *APOC3* nonsense allele (R19X) reported by Pollin et al.,^25^ which presumably decreased APOC3 expression by 50% in heterozygotes, caused a 45% reduction in plasma TG levels. Taken together, these results indicate that the rs2854117 allele has very modest effects on APOC3 expression and plasma TG levels.

Hepatic steatosis is now recognized as one of the adverse metabolic consequences of obesity that constitute metabolic syndrome.^30^ Despite extensive investigation, the mechanistic couplings between the various components of the syndrome have not been fully defined. In cross-sectional studies, the liver fat content has been correlated with indices of insulin resistance.^3^, ^31^, ^32^ These findings are consistent with the proposal that high liver TG levels cause insulin resistance.^31^, ^32^ However, data from humans with genetic variations that cause a primary increase in the liver fat content are incompatible with this view. Mutations in APOB lead to hepatic steatosis but are not associated with increased HOMA-IR^33^ or reduced insulin-mediated glucose disposal.^34^ Similarly, the I148M allele of PNPLA3 has been systematically associated with increased liver TG content but not with insulin resistance as determined by HOMA-IR or euglycemic clamp studies.^4^ Thus, increased liver TG content per se does not lead to insulin resistance.

## Abbreviations

ALT: alanine aminotransferase
APO: apolipoprotein
ARIC: Atherosclerosis Risk in Communities
AST: aspartate aminotransferase
BMI: body mass index
HOMA-IR: homeostatic model assessment of insulin resistance
HTGC: hepatic triglyceride content
MAF: minor allele frequency
MRS: magnetic resonance spectroscopy
NAFLD: nonalcoholic fatty liver disease
PNPLA3: patatin-like phospholipase domain containing 3
ρ: Spearman's rank correlation coefficient
SNP: single-nucleotide polymorphism
TG: triglyceride

## Supplementary material

Supporting Figure 1. Linkage disequilibrium (R2) between single nucleotide polymorphisms in APO A5/A4/C3/A1 gene cluster on chromosome 11q23 in the African-Americans (top), European-Americans (middle) and Hispanics (bottom) in the Dallas Heart Study. Gene and SNP positions correspond to NCBI genome build 34 (July 2003). SNPs were genotyped using a custom oligonucleotide genotyping array (Perlegen Sciences). Correlations were calculated from genotype data of 1,730 African Americans, 996 European Americans and 581 Hispanics. Only common variants (minor allele frequency (MAF) of ≥ 5%) are shown in the correlation map for each ethnic group.

Supporting Figure 2. Linkage disequilibrium (R2) between 41 single nucleotide polymorphisms in the APO A5/A4/C3/A1 gene cluster on chromosome 11q23 in African-Americans (top), European-Americans (bottom) in the ARIC study. Gene and SNP positions correspond to NCBI genome build 36 (March 2006).

Supporting Table 1. Oligonucleotides used for genotyping APOC3 SNPs in the Dallas Heart Study (DHS) population and in Atherosclerosis Risk in Communities (ARIC) study. Supporting Table 2. Clinical characteristics of the Dallas Heart Study participants stratified by APOC3 rs2854117 genotype and ethnicity. Supporting Table 3. Clinical characteristics of the Dallas Heart Study participants stratified by APOC3 rs2854116 genotype and ethnicity. Supporting Table 4. Clinical characteristics of the Dallas Heart Study participants stratified by APOC3 rs2854117 genotype Supporting Table 5. Clinical characteristics of the Dallas Heart Study participants stratified by APOC3 rs2854116 genotype. Supporting Table 6. Clinical characteristics of Atherosclerosis Risk in Communities study (ARIC) participants stratified by APOC3 rs2854117 genotype and ethnicity. Supporting Table 7. Clinical characteristics of Atherosclerosis Risk in Communities study (ARIC) participants stratified by APOC3 rs2854116 genotype and ethnicity. Supporting Table 8. Clinical characteristics of APOC3 wild-type homozygotes (rs2854117 C/C and rs2854116 T/T) compared to carriers of one or more variant alleles (rs2854117 T and rs2854116 C) in Atherosclerosis Risk in Communities study (ARIC) participants, stratified by ethnicity.
