# Supplementary figures and images for: Dissociation Between APOC3 Variants, Hepatic Triglyceride Content and Insulin Resistance
Source: Hepatology. 2011 Feb;53(2):467–74. doi: 10.1002/hep.24072 (PMC3057507; doi:10.1002/hep.24072)

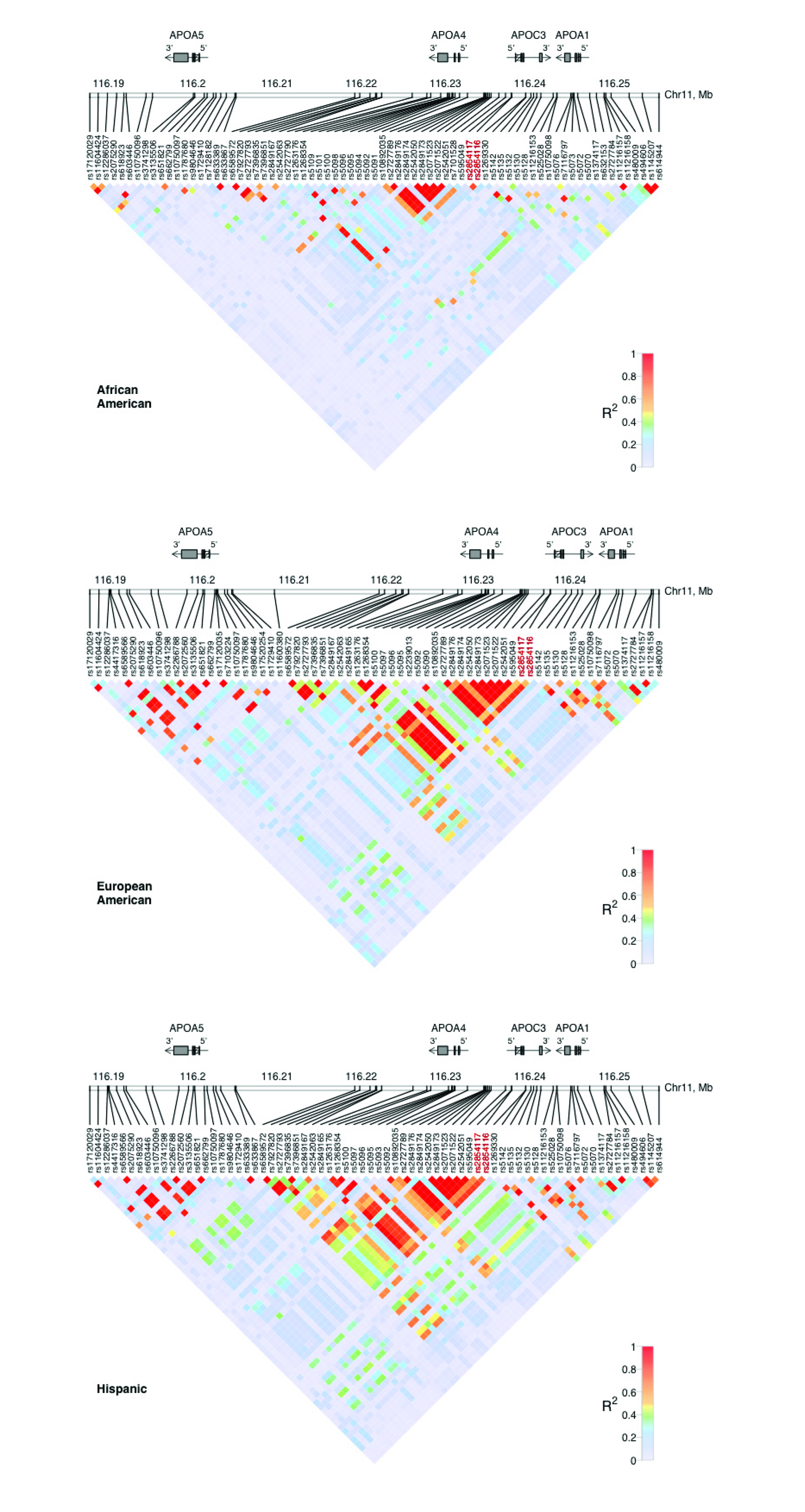

Supplement: Supplementary file 1 [file hep0053-0467-SD1.tif]

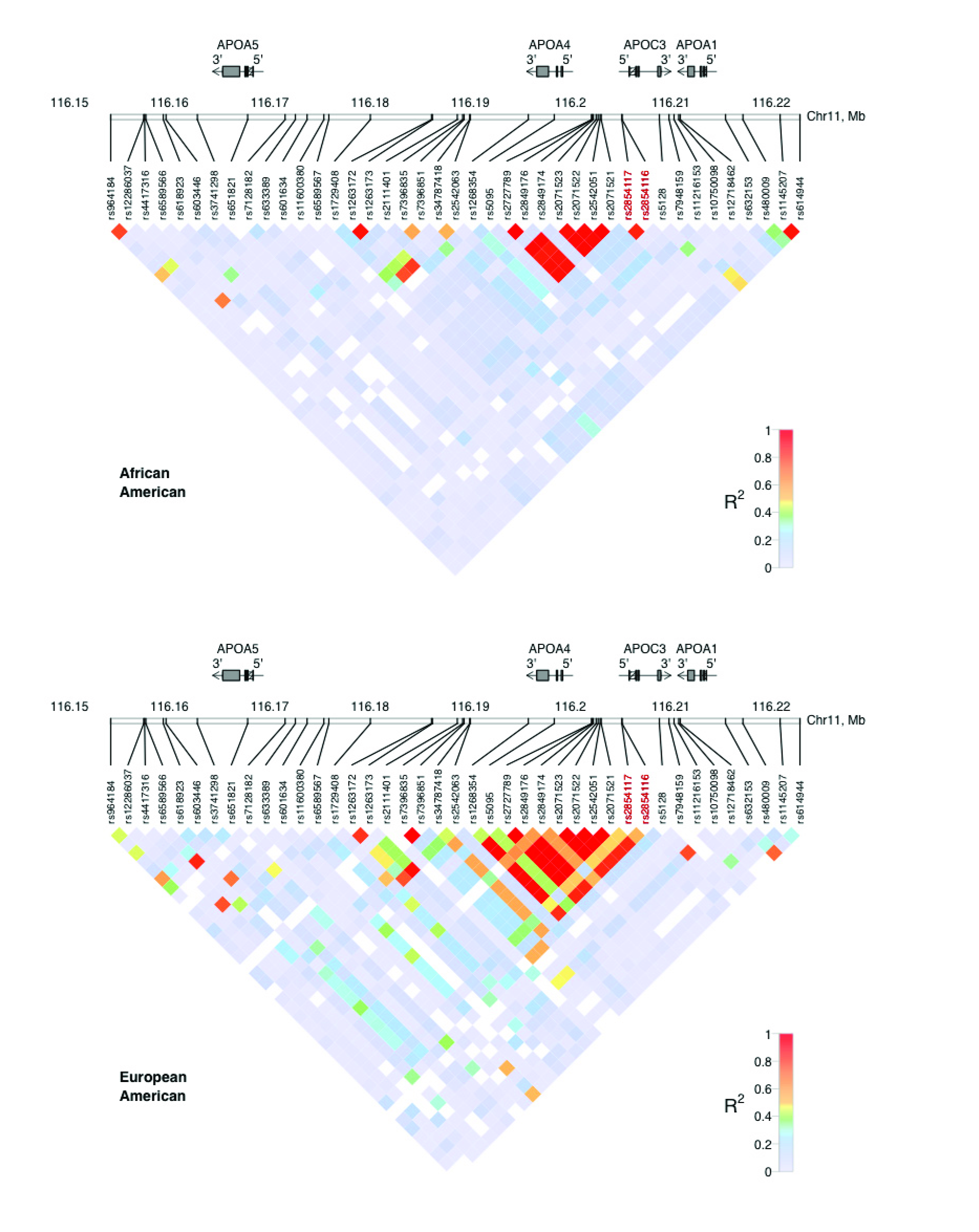

Supplement: Supplementary file 2 [file hep0053-0467-SD2.tif]
