# Supplementary material for: Dissociation Between APOC3 Variants, Hepatic Triglyceride Content and Insulin Resistance
Source: Hepatology. 2011 Feb;53(2):467–74. doi: 10.1002/hep.24072 (PMC3057507; doi:10.1002/hep.24072)
Supplement: Supplementary file 3 [file hep0053-0467-SD3.doc]

| Supporting Table 1. Oligonucleotides used for genotyping APOC3 SNPs in the Dallas Heart Study (DHS) population and in Atherosclerosis Risk in Communities (ARIC) study. | | |
| --- | --- | --- |
| SNP_ID | Forward Primer | Reverse Primer |
| DHS |  |  |
| rs2854117 | GAGAGCTCAGCCCTGTAACCA | CCTGAACACAGCCTGGAGTAGA |
| rs2854116 | CTTGCCGGAGCCACTGAT | TGAGGGGCTTCTTCAGACTTGA |
|  |  |  |
| ARIC |  |  |
| rs2854117 | GGTGTTTGGAGTAAAGGCACAG | AGAGCTCAGCCCTGTAACCA |
| rs2854116 | GGGCTTCTTCAGACTTGAGAACAA | GCCTGGTCTTCTGTGCCTTT |

| Supporting Table 2. Clinical characteristics of the Dallas Heart Study participants stratified by APOC3 rs2854117 genotype and ethnicity. | | | | | | | | | | | | |
| --- | --- | --- | --- | --- | --- | --- | --- | --- | --- | --- | --- | --- |
|  | African Americans | | | | European Americans | | | | Hispanics | | | |
|  | C/C | C/T | T/T | P | C/C | C/T | T/T | P | C/C | C/T | T/T | P |
| N | 152 | 555 | 579 | ^†^0.287 | 479 | 349 | 52 | ^†^0.291 | 202 | 193 | 41 | ^†^0.658 |
| Female/Male | 86/66 | 321/234 | 331/248 |  | 252/227 | 163/186 | 32/20 |  | 103/99 | 117/76 | 29/12 |  |
| Age (years) | 45 ± 9.4 | 44 ± 9.7 | 45.1 ± 9.9 | 0.361 | 45.4 ± 10.1 | 44.3 ± 9.3 | 43.7 ± 9.9 | 0.084 | 40.8 ± 9 | 38.5 ± 8 | 39.7 ± 8.1 | 0.041 |
| BMI (kg/m^2^) | 31.2 ± 7.3 | 31.3 ± 8.2 | 30.9 ± 7.8 | 0.454 | 28.6 ± 6.1 | 28.7 ± 6.4 | 27.7 ± 5.8 | 0.636 | 29.7 ± 5.1 | 30.1 ± 6.6 | 29.9 ± 7.2 | 0.889 |
| Insulin (mIU/L) | 13.8 (8.7-21.8) | 13.1 (8.0-20.7) | 12.9 (7.4-21.1) | 0.484 | 9.8 (6.0-16.6) | 9.9 (6.2-16.1) | 10.7 (6.1-15.9) | 0.314 | 14.0 (8.6-20.1) | 13.7 (7.7-20.4) | 10.7 (7.0-18.5) | 0.137 |
| Glucose (mg/dL) | 90 (83-98) | 89 (83-97) | 90 (84-98) | 0.367 | 91 (84-98) | 90 (83-97) | 91 (84-97) | 0.904 | 96 (87-100) | 93 (87-100) | 94 (87.5-102.5) | 0.952 |
| HOMA-IR (U) | 3.0 (1.8-4.8) | 3.0 (1.7-4.7) | 2.9 (1.6-4.9) | 0.638 | 2.3 (1.3-3.9) | 2.2 (1.3-3.7) | 2.3 (1.3-3.7) | 0.342 | 3.2 (1.9-4.7) | 3.0 (1.7-4.8) | 2.4 (1.5-4.5) | 0.161 |
| TG (mg/dL) | 78 (55.5-114) | 78 (59-111.5) | 84 (62-118.5) | 0.014 | 103 (72-156) | 109 (79-166) | 109 (64-143) | 0.387 | 116 (83-173) | 118 (79-188) | 93 (69-127) | 0.705 |
| HTGC (%) | 3.3 (2.0-5.1) | 3.1 (2.0-4.8) | 3.0 (1.8-4.9) | 0.548 | 3.5 (1.9-6.6) | 3.7 (2.2-6.8) | 2.8 (1.6-4.7) | 0.532 | 4.6 (2.8-11.8) | 4.4 (2.5-9.2) | 3.5 (2.4-6.5) | 0.058 |
| AST (U/L) | 20 (17-23) | 20 (17-26) | 21 (17-27) | 0.4017 | 21 (18-25) | 21 (18-25) | 22 (19-24) | 0.408 | 21 (17-29) | 21 (18-27) | 20 (17-23) | 0.319 |
| ALT (U/L) | 16 (12-23) | 18 (13-26) | 18 (13-26) | 0.214 | 20 (15.5-27) | 21 (15-27) | 18 (16-24) | 0.199 | 21.5 (16-34) | 21 (15-33) | 18 (15-23) | 0.152 |
| Reported numbers are mean±SD for age and BMI, and median (interquartile range) for all other characteristics. P-values were calculated using a linear regression model, as described in the Methods. ^†^P-value for deviation from Hardy-Weinberg equilibrium. Abbreviations: BMI, body mass index; HOMA-IR, homeostatic model assessment of insulin resistance; TG, triglyceride; HTGC, hepatic triglyceride content; AST, aspartate aminotransferase; ALT, alanine aminotransferase. | | | | | | | | | | | | |

| Supporting Table 3. Clinical characteristics of the Dallas Heart Study participants stratified by APOC3 rs2854116 genotype and ethnicity. | | | | | | | | | | | | |
| --- | --- | --- | --- | --- | --- | --- | --- | --- | --- | --- | --- | --- |
|  | African Americans | | | | European Americans | | | | Hispanics | | | |
|  | T/T | T/C | C/C | P | T/T | T/C | C/C | P | T/T | T/C | C/C | P |
| N | 107 | 501 | 643 | ^†^0.489 | 331 | 403 | 120 | ^†^0.942 | 166 | 211 | 63 | ^†^0.840 |
| Female/Male | 59/48 | 287/214 | 371/272 |  | 168/163 | 195/208 | 67/53 |  | 86/80 | 130/81 | 40/23 |  |
| Age (years) | 45.4 ± 10 | 44.2 ± 9.7 | 44.7 ± 9.8 | 0.972 | 45.1 ± 9.9 | 44.5 ± 9.7 | 45.1 ± 9.6 | 0.796 | 40.6 ± 8.8 | 38.8 ± 8.2 | 40.4 ± 9.1 | 0.338 |
| BMI (kg/m^2^) | 31.1 ± 7.3 | 31.4 ± 8.4 | 31 ± 7.7 | 0.451 | 28.8 ± 6.4 | 28.6 ± 6.1 | 28.1 ± 6.3 | 0.327 | 29.5 ± 5.2 | 30.1 ± 6.3 | 30.1 ± 8.5 | 0.751 |
| Insulin (mIU/L) | 14.4 (9.1-22.1) | 13.1 (7.9-20.8) | 12.5 (7.5-20.6) | 0.145 | 10.0 (6.1-16.6) | 9.9 (5.9-16.6) | 10.1 (6.3-15.7) | 0.503 | 13.9 (8.4-20.4) | 13.4 (7.7-20.5) | 9.1 (7.0-17.4) | 0.038 |
| Glucose (mg/dL) | 91 (84-99) | 89 (83-97) | 90 (84-98) | 0.916 | 91 (84-97) | 91 (83-98) | 91 (83-97) | 0.73 | 95 (87-100.5) | 93 (87-99) | 94.5 (88-101) | 0.746 |
| HOMA_IR (U) | 3.2 (1.9-5.2) | 3.0 (1.7-4.8) | 2.8 (1.6-4.7) | 0.19 | 2.3 (1.3-3.9) | 2.2 (1.3-3.9) | 2.2 (1.3-3.7) | 0.513 | 3.2 (1.9-4.8) | 3.0 (1.7-5.0) | 2.1 (1.5-4.3) | 0.048 |
| TG (mg/dL) | 76 (56.5-121) | 79 (60-113) | 82 (60-115) | 0.108 | 107 (75-165) | 106 (74-155) | 101.5 (66-152) | 0.527 | 117 (82-175.5) | 116 (78-179) | 100 (77.5-138) | 0.959 |
| HTGC (%) | 3.7 (2.3-5.5) | 3.1 (2.0-4.8) | 2.9 (1.8-4.7) | 0.043 | 3.7 (1.9-7.4) | 3.5 (2.1-6.5) | 3.2 (2.3-6.9) | 0.755 | 4.4 (2.7-11.8) | 4.4 (2.6-9.0) | 3.8 (2.1-7.4) | 0.122 |
| AST (U/L) | 20 (17-23) | 20 (17-26) | 21 (17-27) | 0.243 | 21 (18-25) | 21 (18-26) | 21 (18-24) | 0.575 | 21 (17-28.5) | 21 (17.5-27) | 21 (17-25) | 0.67 |
| ALT (U/L) | 16 (12-23) | 18 (14-25) | 18 (13-26) | 0.269 | 20 (15-27) | 21 (16-28) | 19 (16-25) | 0.489 | 20.5 (15-33) | 20 (15-33.5) | 19 (15.5-26) | 0.404 |
| Reported numbers are mean±SD for age and BMI, and median (interquartile range) for all other characteristics. P-values were calculated using a linear regression model, as described in the Methods. Abbreviations: BMI, body mass index; HOMA-IR, homeostatic model assessment of insulin resistance; TG, triglyceride; HTGC, hepatic triglyceride content; AST, aspartate aminotransferase; ALT, alanine aminotransferase. ^†^P-value for deviation from Hardy-Weinberg equilibrium. | | | | | | | | | | | | |

| Supporting Table 4. Clinical characteristics of the Dallas Heart Study participants stratified by APOC3 rs2854117 genotype | | | | |
| --- | --- | --- | --- | --- |
|  | C/C | C/T | T/T | P-value |
| N | 833 | 1097 | 672 |  |
| Female/Male | 441/392 | 601/496 | 392/280 |  |
| Age (years) | 44.2 ± 9.9 | 43.1 ± 9.6 | 44.6 ± 9.9 | 0.311 |
| BMI (kg/m^2^) | 29.4 ± 6.2 | 30.3 ± 7.5 | 30.6 ± 7.7 | 0.359 |
| Insulin (mIU/L) | 11.6 (6.6-18.4) | 11.8 (7.4-19.2) | 12.5 (7.1-20.6) | 0.592 |
| Glucose (mg/dL) | 92 (85-99) | 91 (84-98) | 91 (84-98) | 0.424 |
| HOMA-IR (U) | 2.6 (1.5-4.3) | 2.7 (1.6-4.4) | 2.8 (1.5-4.8) | 0.723 |
| TG (mg/dL) | 100 (71-149) | 93 (67-140) | 85 (63-122) | 0.013 |
| HTGC (%) | 3.7 (2.0-7.2) | 3.4 (2.1-6.0) | 3.0 (1.8-5.0) | 0.165 |
| AST (U/L) | 21 (18-26) | 21 (18-26) | 21 (17-26) | 0.831 |
| ALT (U/L) | 20 (15-28) | 19 (14-27) | 18 (13-26) | 0.753 |
| Reported numbers are mean±SD for age and BMI, and median (interquartile range) for all other characteristics. P-values were calculated using a linear regression model, as described in the Methods. Abbreviations: BMI, body mass index; HOMA-IR, homeostatic model assessment of insulin resistance; TG, triglyceride; HTGC, hepatic triglyceride content; AST, aspartate aminotransferase; ALT, alanine aminotransferase. | | | | |

| **Supporting** Table 5. Clinical characteristics of the Dallas Heart Study participants stratified by APOC3 rs2854116 genotype. | | | | |
| --- | --- | --- | --- | --- |
|  | T/T | T/C | C/C | P-value |
| N | 604 | 1115 | 826 |  |
| Female/Male | 313/291 | 612/503 | 478/348 |  |
| Age (years) | 43.9 ± 9.8 | 43.3 ± 9.7 | 44.4 ± 9.8 | 0.59 |
| BMI (kg/m^2^) | 29.4 ± 6.3 | 30.2 ± 7.3 | 30.5 ± 7.6 | 0.284 |
| Insulin (mIU/L) | 11.8 (6.8-18.9) | 11.7 (7.2-19.1) | 11.8 (7.1-19.8) | 0.129 |
| Glucose (mg/dL) | 92 (85-99) | 91 (84-98) | 90.5 (84-98) | 0.902 |
| HOMA-IR (U) | 2.6 (1.5-4.3) | 2.7 (1.6-4.4) | 2.7 (1.6-4.5) | 0.165 |
| TG (mg/dL) | 104 (72-156) | 94 (67-138) | 85 (63-122) | 0.52 |
| HTGC (%) | 3.9 (2.1-8.0) | 3.4 (2.1-6.0) | 3.0 (1.8-5.1) | 0.041 |
| AST (U/L) | 21 (18-25) | 21 (18-26) | 21 (17-26) | 0.719 |
| ALT (U/L) | 20 (15-28) | 19 (14-27) | 18 (13-26) | 0.964 |
| Reported numbers are mean±SD for age and BMI, and median (interquartile range) for all other characteristics. P-values were calculated using a linear regression model, as described in the Methods. Abbreviations: BMI, body mass index; HOMA-IR, homeostatic model assessment of insulin resistance; TG, triglyceride; HTGC, hepatic triglyceride content; AST, aspartate aminotransferase; ALT, alanine aminotransferase. | | | | |

| **Supporting** Table 6. Clinical characteristics of Atherosclerosis Risk in Communities study (ARIC) participants stratified by APOC3 rs2854117 genotype and ethnicity. | | | | | | | | |
| --- | --- | --- | --- | --- | --- | --- | --- | --- |
|  | African Americans | | | | European Americans | | | |
|  | C/C | C/T | T/T | P | C/C | C/T | T/T | P |
| N | 312 | 1328 | 1444 | ^†^ 0.803 | 5410 | 3876 | 645 | ^†^ 0.167 |
| Female/Male | 1 2/3 | 1 310/509 | 1 306/569 | 0.757 | 1 37/198 | 1 105/659 | 313/332 | 0.027 |
| Age (years) | 52.8±5.8 | 53.1±5.9 | 53.1±5.7 | 0.728 | 54.2±5.7 | 54.2±5.7 | 54.4±5.7 | 0.437 |
| BMI (kg/m^2^) | 29.4±5.9 | 28.7±5.9 | 29.2±6.1 | 0.386 | 26.6±4.6 | 26.7±4.6 | 27.0±4.7 | 0.028 |
| Insulin (mIU/L) | 11.3(9.3) | 11.4(9.3) | 11.4(10.3) | 0.203 | 8.26(6.20) | 8.26(6.20) | 8.26(6.20) | 0.575 |
| Glucose(mg/dL) | 99.7±9.8 | 98.9±10.2 | 98.4±10.0 | 0.023 | 98.9±9.2 | 98.8±9.2 | 99.2±9.1 | 0.062 |
| HOMA-IR (U) | 2.74(2.34) | 2.66(2.58) | 2.80(2.58) | 0.411 | 2.05(1.79) | 2.06(1.83) | 2.08(1.83) | 0.785 |
| TG (mg/dL) | 90(50) | 90(57) | 92(56) | 0.808 | 109(75) | 112(79) | 117(84) | 0.001 |
| Reported numbers are mean±SD or median (interquartile range). P-values were calculated using a linear regression model, adjusted for age, sex and BMI (where appropriate). ^†^P-value for deviation from Hardy-Weinberg equilibrium. Abbreviations: BMI, body mass index; HOMA-IR, homeostatic model assessment of insulin resistance; TG, triglyceride. | | | | | | | | |

| **Supporting** Table 7. Clinical characteristics of Atherosclerosis Risk in Communities study (ARIC) participants stratified by APOC3 rs2854116 genotype and ethnicity. | | | | | | | | |
| --- | --- | --- | --- | --- | --- | --- | --- | --- |
|  | African Americans | | | | European Americans | | | |
|  | T/T | T/C | C/C | P | T/T | T/C | C/C | P |
| N | 246 | 1276 | 1520 | ^†^0.356 | 3987 | 4577 | 1311 | ^†^0.983 |
| Female/Male | 1 64/91 | 1 294/491 | 1 78/151 | 0.642 | 1 204/971 | 1 104/789 | 1 15/211 | 0.107 |
| Age (years) | 52.9±5.7 | 53.0±5.8 | 53.1±5.7 | 0.691 | 54.1±5.7 | 54.2±5.7 | 54.2±5.6 | 0.685 |
| BMI (kg/m^2^) | 29.6±5.9 | 28.8±6.0 | 29.1±6.0 | 0.998 | 26.6±4.7 | 26.7±4.6 | 26.7±4.5 | 0.332 |
| Insulin (mIU/L) | 11.4(8.3) | 10.3(9.3) | 11.4(10.3) | 0.232 | 8.26(6.20) | 8.26(6.20) | 8.26(6.20) | 0.726 |
| Glucose(mg/dL) | 99.7±9.4 | 98.5±10.2 | 98.6±10.0 | 0.273 | 98.3±9.0 | 98.7±9.1 | 99.0±9.1 | 0.042 |
| HOMA_IR (U) | 2.86(2.26) | 2.63(2.57) | 2.73(2.56) | 0.351 | 2.02(1.76) | 2.08(1.85) | 2.10(1.82) | 0.534 |
| TG (mg/dL) | 90(62) | 89(56) | 91(56) | 0.983 | 110(76) | 111(79) | 114(79) | 0.056 |
| Reported numbers are mean±SD or median (interquartile range). P-values were calculated using a linear regression model, adjusted for age, sex and BMI (where appropriate). ^†^P-value for deviation from Hardy-Weinberg equilibrium. Abbreviations: BMI, body mass index; HOMA-IR, homeostatic model assessment of insulin resistance; TG, triglyceride. | | | | | | | | |

| **Supporting T**able 8. Clinical characteristics of APOC3 wild-type homozygotes (rs2854117 C/C and rs2854116 T/T) compared to carriers of one or more variant alleles (rs2854117 T and rs2854116 C) in Atherosclerosis Risk in Communities study (ARIC) participants, stratified by ethnicity. | | | | | | |
| --- | --- | --- | --- | --- | --- | --- |
|  | African Americans | | | European Americans | | |
|  | Wild-type Homozygotes | Variant-Allele Carriers | P | Wild-type Homozygotes | Variant-Allele Carriers | P |
| N | 231 | 2752 |  | 3841 | 5958 |  |
| Female/Male | 1 3/4 | 1 50/241 | 0.440 | 1 50/241 | 1 88/705 | 0.089 |
| Age (years) | 52.0±5.7 | 53.1±5.8 | 0.561 | 54.1±5.7 | 54.2±5.7 | 0.358 |
| BMI (kg/m^2^) | 29.6±5.8 | 29.0±6.0 | 0.233 | 26.5±4.7 | 26.7±4.6 | 0.079 |
| Insulin (mIU/L) | 11.3(8.3) | 11.4(10.3) | 0.567 | 8.26(6.20) | 8.26(6.20) | 0.820 |
| Glucose (mg/dL) | 99.7±9.4 | 98.6±10.1 | 0.176 | 98.3±8.9 | 98.8±9.1 | 0.035 |
| HOMA-IR (U) | 2.89(2.11) | 2.69(2.59) | 0.765 | 2.03(1.76) | 2.08(1.85) | 0.603 |
| TG (mg/dL) | 90.5(52) | 90(56) | 0.877 | 110(76) | 111(79) | 0.381 |
| Reported numbers are mean±SD or median (interquartile range). P-values were calculated using a linear regression model, adjusted for age, sex and BMI (where appropriate). ^†^P-value for deviation from Hardy-Weinberg equilibrium. Abbreviations: BMI, body mass index; HOMA-IR, homeostatic model assessment of insulin resistance; TG, triglyceride. | | | | | | |
